# Blend of Cinnamaldehyde, Eugenol, and Capsicum Oleoresin Improved Rumen Health of Lambs Fed High-Concentrate Diet as Revealed by Fermentation Characteristics, Epithelial Gene Expression, and Bacterial Community

**DOI:** 10.3390/ani13101663

**Published:** 2023-05-17

**Authors:** Wenwen Wang, Yuan Wang, Tao Guo, Chang Gao, Yi Yang, Lei Yang, Zhiwei Cui, Jinju Mao, Na Liu, Xiaoping An, Jingwei Qi

**Affiliations:** 1 College of Animal Science, Inner Mongolia Agricultural University, Hohhot 010018, China; 2 Inner Mongolia Herbivorous Livestock Feed Engineering and Technology Research Center, Hohhot 010018, China; 3 Key Laboratory of Smart Animal Husbandry at Universities of Inner Mongolia Automomous Region, Hohhot 010018, China

**Keywords:** plant extracts, microbiota, rumen barrier, apoptosis, inflammation, high-concentrate diet, lamb

## Abstract

**Simple Summary:**

High-concentrate diets can damage the barrier function of the rumen epithelium and lead to inflammation, which may result in digestive and metabolic disorders and growth retardation in lambs. The phenolic phytonutrients, such as cinnamaldehyde, eugenol, and capsicum oleoresin, have been suggested to act as rumen modifiers. Our results show that a blend of cinnamaldehyde, eugenol, and capsicum oleoresin (CEC) supplementation improved the growth performance and rumen health of lambs fed a high-concentrate diet, which could be due to reducing inflammation and apoptosis, protecting barrier function, and modulating the bacterial community.

**Abstract:**

We investigated the effects of CEC on the fermentation characteristics, epithelial gene expression, and bacterial community in the rumen of lambs fed a high-concentrate diet. Twenty-four 3-month-old female crossbred lambs with an initial body weight of 30.37 ± 0.57 kg were randomly allocated to consume a diet supplemented with 80 mg/kg CEC (CEC) or not (CON). The experiment consisted of a 14 d adaptation period and a 60 d data collection period. Compared with the CON group, the CEC group had higher ADG, epithelial cell thickness, ruminal butyrate proportion, and lower ammonia nitrogen concentration. Increases in the mRNA expression of *Occludin* and *Claudin-4*, as well as decreases in the mRNA expression of apoptotic protease activating factor-1 (*Apaf-1*), cytochrome c (*Cyt-C*), *Caspase-8*, *Caspase-9*, *Caspase-3*, *Caspase-7*, and toll-like receptor 4 (*TLR4*), were observed in the CEC group. Moreover, CEC treatment also decreased the concentration of IL-1β, IL-12, and TNF-α. Supplementation with CEC altered the structure and composition of the rumen bacterial community, which was indicated by the increased relative abundances of Firmicutes, Synergistota, *Rikenellaceae_RC9_gut_group*, *Olsenella*, *Schwartzia*, *Erysipelotrichaceae_UCG-002*, *Lachnospiraceae_NK3A20_group*, *Acetitomaculum*, *[Eubacterium]_ruminantium_group*, *Prevotellaceae_UCG-004*, *Christensenellaceae_R-7_group*, *Sphaerochaeta*, *Pyramidobacter*, and *[Eubacterium]_eligens_group*, and the decreased relative abundances of Acidobacteriota, Chloroflexi, Gemmatimonadota, and *MND1*. Furthermore, Spearman correlation analysis revealed that the altered rumen bacteria were closely correlated with rumen health-related indices. Dietary CEC supplementation improved growth performance, reduced inflammation and apoptosis, protected barrier function, and modulated the bacterial community of lambs fed a high-concentrate diet.

## 1. Introduction

To shorten the fattening time and meet the increasing demand for mutton, lambs are fed high-concentrate feeds instead of fiber-rich forages. However, these dietary transitions affect chewing behavior and rumen buffering, cause an increase in immunogenic compounds and volatile fatty acids (VFA), and then alter ruminal fermentation and microbiota composition [1]. Moreover, these negative effects can damage the barrier function of the rumen epithelium and lead to inflammation, which may result in digestive and metabolic disorders and growth retardation in lambs [2,3]. Numerous preventive strategies have been suggested to maintain rumen health under low ruminal pH conditions, including supplementation with phytonutrients, thiamine, and probiotics [4].

The phenolic phytonutrients are plant-derived bioactive compounds known to possess antioxidant, antimicrobial, and anti-inflammatory effects and are often present in the diet of ruminants [5]. Some phenolic phytonutrients, such as cinnamaldehyde, eugenol, and capsicum oleoresin, have been suggested to act as rumen modifiers. In a previous in vitro study, capsicum oil, eugenol, and cinnamaldehyde additions to diluted ruminal fluid with a 50:50 forage:concentrate diet affected ruminal fermentation with an increase in ruminal pH, the proportion of propionate and butyrate, as well as a decrease in the proportion of acetate and the concentration of ammonia nitrogen [6]. In addition, a blend of cinnamaldehyde, eugenol, carvacrol, and capsicum oleoresin improved microbial protein synthesis and reduced methane emissions and protozoa counts in sheep [7]. However, little information is available on the effects of a blend of cinnamaldehyde, eugenol, and capsicum oleoresin (CEC) on epithelial gene expression and the microbial community in the rumen of lambs. Our hypothesis states that supplementation with CEC will reduce the negative effects of high-concentrate feeding by manipulating microbiota, improving barrier function, alleviating inflammation and apoptosis of the rumen, and then enhancing growth performance. Consequently, the objective of this study was to assess the effects of CEC on growth performance, rumen morphology, fermentation characteristics, epithelium-associated microbiota, cytokine contents, and gene expression of apoptosis and tight junction proteins in lamb.

## 2. Materials and Methods

### 2.1. Animal Ethics Statement

The current study approval for experimental protocols on animals was provided by the Animal Care and Use Committee of Inner Mongolia Agricultural University ([2020]069). All experimental protocols on animals, including euthanasia, sample collection, and carcass disposal procedures, were in strict accordance with the requirements of the Ethics Procedures and Guidelines of the People’s Republic of China.

### 2.2. Feed Additive

The commercial blend of cinnamaldehyde, eugenol, and capsicum oleoresin (XTract 7065) was provided by Pancosma (Shanghai, China) Feed Additives Co. Ltd. (Shanghai, China). The levels of active ingredients in this microencapsulated product guaranteed by the manufacturer were 5.5% cinnamaldehyde, 9.5% eugenol, and 3.5% capsicum oleoresin.

### 2.3. Experimental Design, Animals and Diets

Twenty-four 3-month-old female crossbred lambs (Dorper × Small Tail Han Sheep) with an initial body weight of 30.37 ± 0.57 kg were randomly divided into two groups, with 12 lambs in each group. The lambs in the control group (CON) were fed a basal diet, whereas the lambs in the experimental group (CEC) were provided the same basal diet supplementation with 80 mg/kg CEC for 60 d. The ingredients and nutritional composition of the basal are presented in Table 1. The added dose of CEC was based on our previous study, wherein 80 mg/kg CEC improved growth performance and nutrient digestion in growing ewes [8].

The lambs were housed in separate pens (two lambs in one pen) with facilities for individual feeding and watering in a naturally ventilated barn with windows. The lambs were fed at 08:00 and 17:00 and had free access to freshwater during the trial period.

### 2.4. Sample Collection

At the end of the experiment, twelve lambs (one lamb in each pen) were selected to be slaughter from each pen using Halal methods in the local abattoir. The lambs were slaughtered at the end of the trial, 2 h following the last feeding. The time lag between slaughtering and ruminal fluid sampling was ~25 min. After slaughter, rumen content was individually collected into 2.5 mL sterile polypropylene centrifuge tubes, snapped frozen in liquid nitrogen, and kept at −80 °C for 16S ribosomal RNA-based taxonomic analysis. Approximately 0.2 L of ruminal fluid was collected from each lamb and strained through 4 layers of cheesecloth for ruminal fermentation parameter measurements. Rumen tissue (15 g) was collected into 5 mL sterile polypropylene centrifuge tubes and transferred into liquid nitrogen for ELISA and quantitative real-time PCR analysis. For the analysis of histomorphology, rumen tissue (1.5 by 1.5 cm^2^) samples were fixed in 4% paraformaldehyde.

### 2.5. Growth Performance

The provided feed and rejections were recorded daily during the experiment for the daily dry matter intake (DMI) calculation. On days 0 and 60, the shrunk body weight of lambs (lambs were denied access to water and feed for 12 h) was determined to calculate the average daily gain (ADG) and feed efficiency (DMI:ADG).

### 2.6. Histomorphological Analysis

The rumen morphology was assessed using the method previously described [9]. Briefly, rumen samples were fixed with a 4% paraformaldehyde solution, dehydrated in a graded series of ethanol, and then embedded in paraffin, sectioned, and dyed with hematoxylin and eosin for histological observation. The papillae width, papillae length, muscle layer thickness, and epithelial cell thickness of each rumen were measured using a Motic BA610 microscope equipped with a digital camera (Motic China Group Co., Ltd., Xiamen, China) and Motic DS Assistant Lite morphological analysis software (version: 3.0).

### 2.7. Ruminal Fermentation Parameters

The pH value of the ruminal fluid was measured instantly after collection via a pH meter (Starter 2100, Ohaus Corp., Parsippany, NY, USA). The ruminal VFA concentration was measured with a gas chromatograph (Clarus 680, PerkinElmer, Inc., Waltham, MA, USA) using an Elite-FFAP column (30 m in length with a 0.25 mm i.d.) as described [10]. The concentration of ammonia nitrogen in the ruminal fluid was determined with the colorimetric method as previously described [11].

### 2.8. The Concentration of Cytokines Analysis

The interleukin-1β (IL-1β), interleukin-6 (IL-6), interleukin-12 (IL-12), interleukin-10 (IL-10), tumor necrosis factor-α (TNF-α), and interferon-γ (INF-γ) in rumen tissue were measured using the commercial kits (Wuhan ColorfulGene Biological Technology Co., Ltd., Wuhan, China) according to the manufacturer’s instructions. The intra-assay and inter-assay coefficients of variation of the assay kits were 9.0% and 15.0%, respectively.

### 2.9. Quantitative Real-Time PCR Analysis

As previously described [12], the total RNA was extracted from rumen tissue using the TRIzol reagent (Invitrogen, Waltham, MA, USA). RNA purity was determined by DU 640 UV spectrophotometer detection (Beckman Coulter Inc., USA), and the OD260:OD280 ratio ranged from 1.8 to 2.0 in all samples. The RNA integrity was analyzed by electrophoresis on 1% agarose gels. The complementary DNA (cDNA) was synthesized using SuperScript III First-Strand Synthesis SuperMix for qPCR (Invitrogen Life Technologies, Waltham, MA, USA), following the manufacturer’s instructions. The qPCR was performed on a LightCycler 480 Real-Time PCR System (Roche Diagnostics, Indianapolis, IN, USA). Oligonucleotide primers were used to detect the expression of the target genes and a reference gene (β-actin) using the SYBR Green system (Roche Diagnostics GmbH, Mannheim, Germany). The reaction mixture (20 μL) included 10 μL of FS Universal SYBR Green Master Mix, 0.5 μL of forward primer, 0.5 μL of reverse primer, 1 μL of cDNA, and 8 μL of diethylpyrocarbonate-treated water. The amplification protocol was as follows: 95 °C for 1 min per cycle, followed by 40 cycles of 95 °C for 15 s and 63 °C for 25 s. The relative mRNA expression of the target genes was determined using the 2^−ΔΔCt^ method, and data for each target transcript were normalized to the control rats (1.0). The primer details are listed in Table 2.

### 2.10. 16S Ribosomal RNA-Based Taxonomic Analysis

Total DNA from the rumen content samples was extracted using the QIAamp DNA Stool Mini Kit (QIAGEN, Hilden, Germany) according to the manufacturer’s protocols. Additionally, 16S rRNA genes were amplified by PCR from the genomic DNA samples using specific primers for the V4 region of the bacterial 16S rRNA. Amplicon libraries for all samples were quantified by the Qubit 2.0 Fluorometer (Thermo Fisher Scientific, Waltham, MA, USA) and sequenced on a NovaSeq6000 platform by Novogene Bioinformatics Technology Co., Ltd. (Beijing, China) to generate 2 × 250 bp paired-end reads. The singletons and chimeras were removed, and then tags were clustered into the operational taxonomic unit (out) using UPARSE (V 7.0.1001, http://www.drive5.com/uparse/, accessed on March 2022) at 97% similarity. Afterward, the representative sequences of the OTUs were annotated using the SSUrRNA database (SILVA138, http://www.arb-silva.de/, accessed on March 2022). The alpha and beta diversity were analyzed by QIIME. Linear discriminant analysis coupled with effect size (LEfSe) was conducted to identify bacterial taxa differentially represented among the groups at various taxonomy levels. An LDA effect size of more than 3 was used as a threshold for the LEfSe analysis. The obtained raw paired-end reads were deposited in the NCBI Sequence Read Archive (Bioproject: PRJNA912559).

### 2.11. Statistical Analysis

The data were analyzed by SAS 9.2 (SAS Institute, Gary, NC, USA) in a completely randomized design. Individual lambs served as the experimental unit. The *t*-test was used to analyze the remaining data, and the results were expressed as the least squares mean and SEM. A non-parametric test was employed to assess the bacterial data. The Wilcoxon rank sum test was used for multi-group independent samples, and the results are shown as the mean ± SD. The correlations between altered rumen bacteria and rumen health-related indices, which were significantly affected by CEC treatments, were demonstrated by Spearman’s correlation analysis. Values *p* ≤ 0.05 were taken to indicate significance, and 0.05 < *p* ≤ 0.10 was taken as an indication of tendency.

## 3. Results

### 3.1. Growth Performance

The initial body weight, final body weight, DMI, and DMI/ADG were not affected by CEC treatment (*p* > 0.05; Table 3). However, ADG was improved (*p* = 0.02) in response to the infusion of CEC. The CEC supplementation showed a decreased trend (*p* = 0.10) for DMI/ADG in lambs compared with the CON group.

### 3.2. Rumen Morphology

The epithelial cell thickness increased after an oral infusion of CEC (*p* = 0.01; Table 4). However, no considerable differences were found in ruminal papillae length, papillae width, and muscle layer thickness between the control and CEC groups (*p* > 0.05; Table 4).

### 3.3. Ruminal Fermentation Parameters

No significant difference was observed in rumen pH value between the CON and CEC groups (*p* > 0.05; Table 5). The concentration of ammonia nitrogen was decreased (*p* = 0.05; Table 5) in response to the infusion of CEC. Supplementation with CEC increased (*p* = 0.04) butyrate proportion, showed a decreased trend (*p* = 0.10) for propionate proportion, and had no effect on the other ruminal fermentation parameters (*p* > 0.05).

### 3.4. Tight Junction Proteins

**Figure 1:**
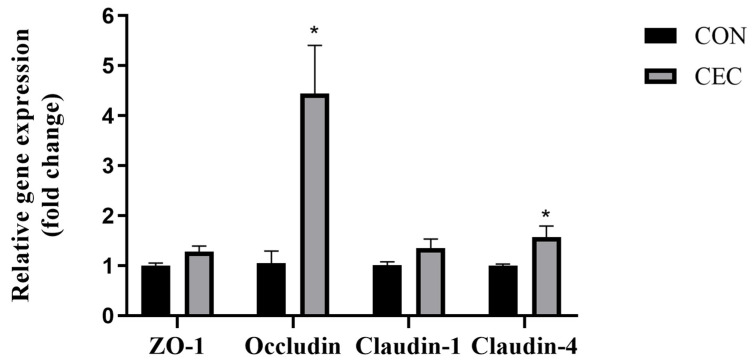
Effects of CEC supplementation on the mRNA expression of tight junction proteins. *ZO-1* = zonula occludens-1; CON = control diet; CEC = supplemented with an 80 mg/kg blend of cinnamaldehyde, eugenol, and capsicum oleoresin. Data are expressed as mean ± SEM. Significant differences are indicated as * *p* ≤ 0.05.

Compared to the CON group, the mRNA expression of *Occludin* and *Claudin-4* was enhanced in the CEC group (*p* < 0.05; Figure 1). In addition, there was no difference between the control and CEC groups regarding zonula occludens-1 (*ZO-1*) and *Claudin-1* expression levels (*p* > 0.05; Figure 1).

### 3.5. Apoptosis Related Genes

**Figure 2:**
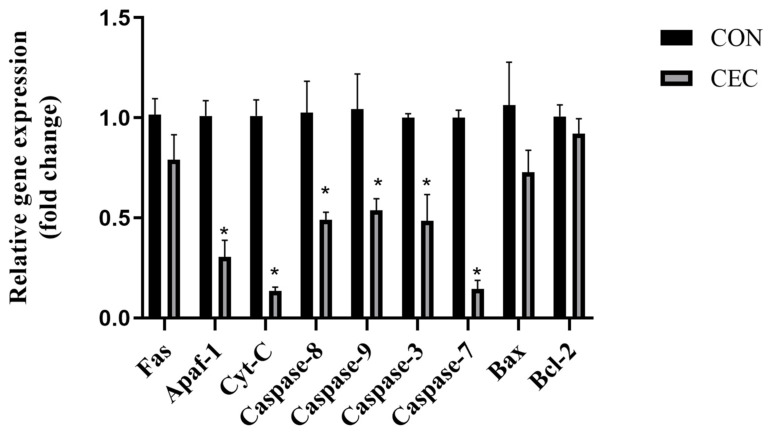
Effects of CEC supplementation on the mRNA expression of apoptosis-related genes. *Apaf-1* = Apoptotic protease activating factor-1; *Cyt-C* = cytochrome c; *Bax* = B-cell lymphoma-2 associated X protein; *Bcl-2* = B-cell lymphoma-2; CON = control diet; CEC = supplemented with an 80 mg/kg blend of cinnamaldehyde, eugenol, and capsicum oleoresin. Data are expressed as mean ± SEM. Significant differences are indicated as * *p* ≤ 0.05.

The mRNA expression of apoptotic protease activating factor-1 (*Apaf-1*), cytochrome c (*Cyt-C*), *Caspase-8*, *Caspase-9*, *Caspase-3*, and *Caspase-7* had a significant decrease in the CEC group compared with the CON group (*p* < 0.05; Figure 2). However, the CEC treatment had no significant effect on the mRNA expression of *Fas*, B-cell lymphoma-2 (*Bcl-2*), and B-cell lymphoma-2 associated X protein (*Bax*) (*p* > 0.05; Figure 2).

### 3.6. Cytokine and Toll-like Receptor 4 (TLR4)

**Figure 3:**
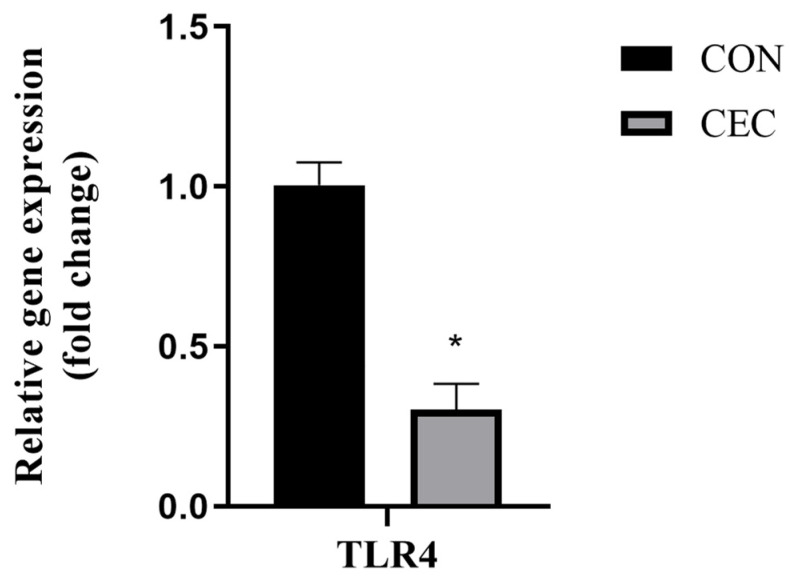
Effects of CEC supplementation on the mRNA expression of *TLR4*. *TLR4* = toll-like receptor 4; CON = control diet; CEC = supplemented with an 80 mg/kg blend of cinnamaldehyde, eugenol, and capsicum oleoresin. Data are expressed as mean ± SEM. Significant differences are indicated as * *p* ≤ 0.05.

Compared to the CON group, the concentrations of IL-1β, IL-12, and TNF-α were decreased in the CEC group (*p* < 0.05; Table 6). Meanwhile, the mRNA expression of *TLR4* was reduced after an oral infusion of CEC (*p* > 0.05; Figure 3). However, CEC treatment had no effect on the other cytokines (*p* > 0.05; Table 6).

### 3.7. Rumen Bacteria Structure and Composition

**Figure 4:**
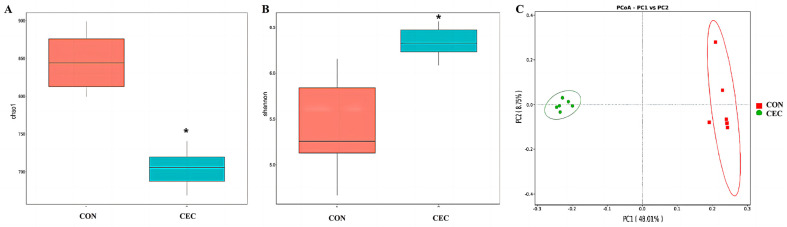
(**A**–**C**) Effects of CEC supplementation on the alpha and beta diversity of the rumen bacterial community in lambs. Analysis of the alpha and beta diversity via (**A**) Chao1 index, (**B**) Shannon index, and (**C**) principal coordinates analysis (PCoA) of unweighted UniFrac distances, respectively. CON = control diet; CEC = supplemented with an 80 mg/kg blend of cinnamaldehyde, eugenol, and capsicum oleoresin. Significant difference: * *p* ≤ 0.05 by Wilcoxon rank sum test.

We selected 1 index of richness and 2 indices of diversity to evaluate the rumen bacteria of lambs. For alpha diversity, supplementation with CEC decreased the Chao1 index but increased the Shannon index (Figure 4A,B). For beta diversity analysis, PCoA based on unweighted UniFrac distances was performed (Figure 4C). The addition of CEC resulted in a clear separation of the rumen bacteria at the OTU level.

**Figure 5:**
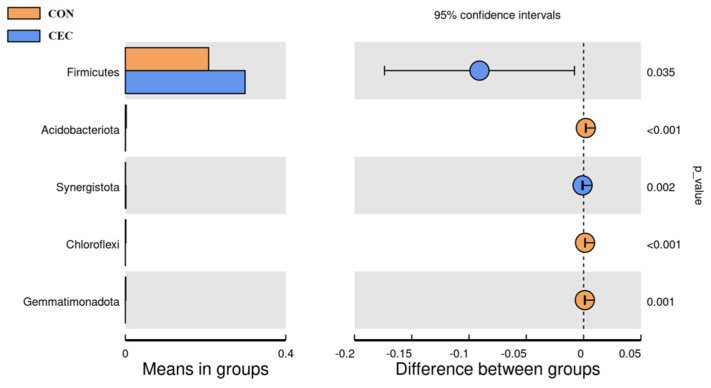
Bacterial taxa differences between the CON and CEC groups at the phylum level. CON = control diet; CEC = supplemented with an 80 mg/kg blend of cinnamaldehyde, eugenol, and capsicum oleoresin.

**Figure A1:**
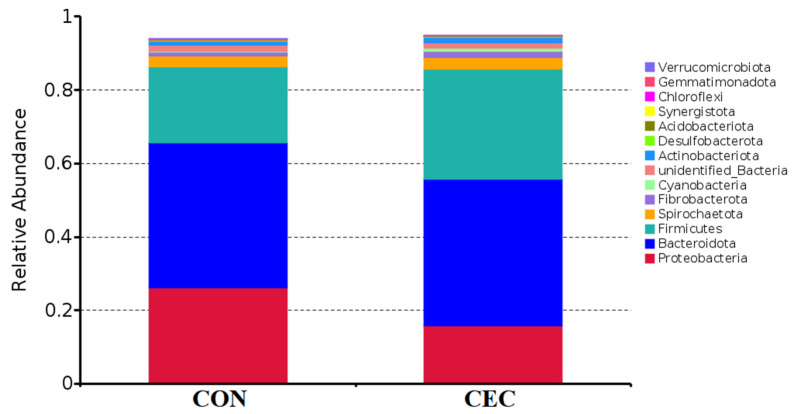
The relative abundances of the 10 most abundant phyla. CON = control diet; CEC = supplemented with an 80 mg/kg blend of cinnamaldehyde, eugenol, and capsicum oleoresin.

As shown in Appendix Figure A1, Bacteroidetes (39.43% vs. 39.95%), Firmicutes (20.76% vs. 29.85%), and Proteobacteria (26.22% vs. 15.88%) were the three predominant phyla in the CON and CEC groups. In addition, there was a substantial increase in Firmicutes and Synergistota (0.04% vs. 0.13%) relative abundance in the CEC group, whereas the relative abundance of Acidobacteriota (0.23% vs. 0.04%), Chloroflexi (0.14% vs. 0.01%), and Gemmatimonadota (0.11% vs. 0.003%) decreased in the CEC group (*p* < 0.05; Figure 5).

**Figure 6:**
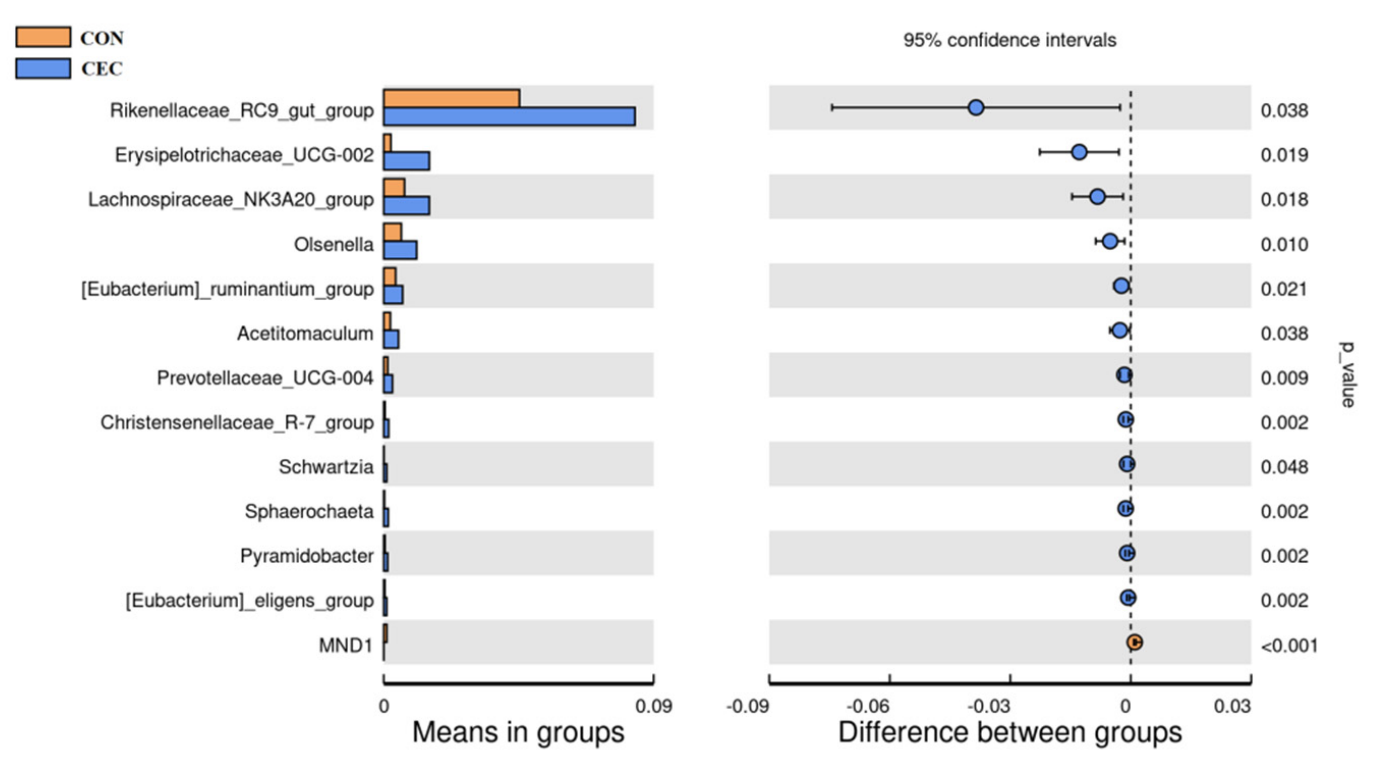
Bacterial taxa differences between CON and CEC groups at the genus level. CON = control diet; CEC = supplemented with an 80 mg/kg blend of cinnamaldehyde, eugenol, and capsicum oleoresin.

At the genus level (Appendix Table A1), we only list the top 59 bacterial genera whose relative abundance was greater than 0.1% in at least one group. Lambs in the CEC group had a higher relative abundance of *Rikenellaceae_RC9_gut_group* (4.53% vs. 8.38%), *Olsenella* (0.58% vs. 1.10%), *Schwartzia* (0.01% vs. 0.10%), *Erysipelotrichaceae_UCG-002* (0.24% vs. 1.52%), *Lachnospiraceae_NK3A20_group* (0.69% vs. 1.52%), *Acetitomaculum* (0.22% vs. 0.49%), *[Eubacterium]_ruminantium_group* (0.40% vs. 0.63%), *Prevotellaceae_UCG-004* (0.13% vs. 0.29%), *Christensenellaceae_R-7_group* (0.04% vs. 0.16%), *Sphaerochaeta* (0.02% vs. 0.15%), *Pyramidobacter* (0.04% vs. 0.13%), and *[Eubacterium]_eligens_group* (0.04% vs. 0.10%) compared to lambs in the CON group (*p* < 0.05; Figure 5). However, CEC treatment decreased the relative abundance of MND1 (0.10% vs. 0.002%) (*p* < 0.05; Figure 6).

**Figure 7:**
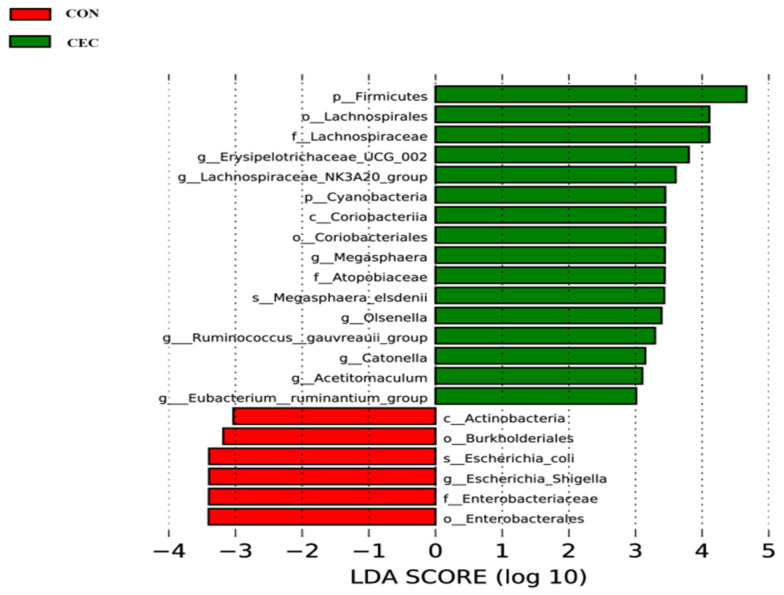
Linear discriminant analysis (LDA) coupled with effect size (LEfSe) analysis was performed to identify the bacterial taxa differentially represented in the CON and CEC groups at different taxonomy levels. CON = control diet; CEC = supplemented with an 80 mg/kg blend of cinnamaldehyde, eugenol, and capsicum oleoresin.

The LEfSe approach was performed to identify the specific bacterial taxa that differed between the CON and CEC groups at different taxonomic levels (Figure 7). Lambs in the CON group enriched Actinobacteria at the class level, Burknolderiales and Enterobacterales at the order level, Enterobacteriaceae at the family level, *Escherichia_Shigella* at the genus level, and *Escherichia_coli* at the species level. Lambs in the CEC group enriched Firmicutes and Cyanobacteria at the phylum level, Coriobacteriia at the class level, Lachnospirales and Coriobacteriales at the order level, Lachnospiraceae and Atopobiaceae at the family level, *Erysipelotrichaceae_UCG-002*, *Lachnospiraceae_NK3A20_group*, *Megasphaera*, *Olsenella*, *Ruminococcus_gauvreauii_group*, *Catonella*, *[Eubacterium]_ruminantium_group,* and *Acetitomaculum* at the genus level, and *Megasphaera_elsdenii* at the species level.

### 3.8. Correlation between Altered Rumen Bacteria and Rumen Health Related Indices

**Figure 8:**
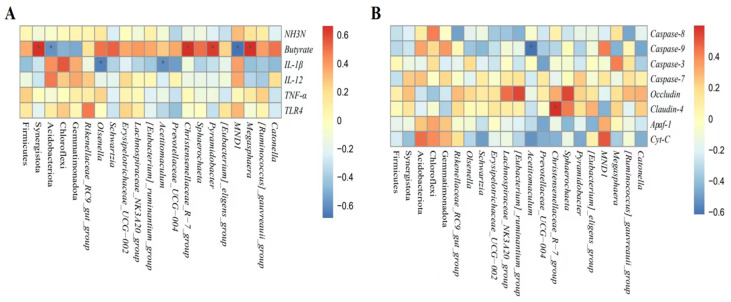
(**A**,**B**) Correlation between altered rumen bacteria and rumen health-related indices. (**A**) Ruminal fermentation and inflammation parameters. (**B**) Tight junction proteins and apoptosis related genes parameters. NH_3_-N = ammonia nitrogen; IL-1β = interleukin-1β; IL-12 = interleukin-12; TNF-α = tumor necrosis factor-α; *TLR4* = toll-like receptor 4; *Apaf-1* = Apoptotic protease activating factor-1; *Cyt-C* = cytochrome c.

As shown in Figure 8A,B, the relative abundances of *Megasphaera*, *Pyramidobacter*, *Christensenellaceae_R-7_group,* and *Synergistota* were positively correlated with butyrate proportion (*p* < 0.05). Meanwhile, the relative abundances of *Christensenellaceae_R-7_group* were positively correlated with the mRNA expression of *Claudin-4* (*p* < 0.05). However, the relative abundances of *MND1* and Acidobacteriota were negatively correlated with butyrate proportion (*p* < 0.05). In addition, the relative abundances of *Acetitomaculum* and *Olsenella* were negatively correlated with IL-1β concentration and *Caspase-9* mRNA expression (*p* < 0.05).

## 4. Discussion

The current study showed that CEC supplementation improved ADG and had a decreased trend for DMI/ADG in lambs. This result reaffirms previous reports by An et al. [8]. In modern sheep farming, high-concentrate diets can easily result in the accumulation of VFA produced from microbial fermentation and a depressed ruminal pH [13]. In the present experiment, ruminal pH ranged from 5.22 to 5.34, which is due to a high-concentration diet. The ruminal pH was not affected by CEC supplementation, as expected, with no changes in total VFA observed. Consistent with our study, Geraci et al. found that CEC did not alter the ruminal pH or total VFA concentration of cattle [14]. However, the addition of CEC decreased the concentration of ammonia nitrogen and increased the butyrate proportion. Cardozo et al. and Geraci et al. found a reduction in ruminal ammonia with the same CEC blend [14,15]. Much (up to 50% of the total) of the ruminal ammonia nitrogen is produced by hyper ammonia-producing bacteria [16]. However, the phenolic components can inhibit hyper ammonia-producing bacteria populations [17,18]. The low ammonia nitrogen concentration in CEC lambs may be responsible for the inhibition of hyper ammonia-producing bacteria by CEC (rich in phenolic components). Previous studies have confirmed that CEC treatment enhanced molar proportions of butyrate in the rumen of Holstein steers [19]. Butyrate is the preferred energy source for rumen epithelial and accelerates ruminal epithelium growth and maturation [20]. Our study revealed an increase in epithelial cell thickness as a result of CEC feeding. Herein, we speculate that the supplementation of CEC promoted the fermentation to produce more butyrate and stimulated the growth of the ruminal epithelium.

Tight junction proteins such as *Occludin*, *Claudin-1*, *Claudin-4*, and *ZO-1* act as vital components in modulating epithelial barrier function [21]. It is well known that acidotic luminal pH damages the epithelial barrier and decreases tight junction protein expression in sheep [22]. Upregulation of *Occludin* and *Claudin-4* mRNA expression was observed in the rumen epithelium of lambs supplemented with CEC. To our knowledge, this is the first study to observe rumen tight junction proteins with CEC supplementation. As a kind of phenolic phytonutrient, phloretin increased occludin mRNA expression and *Claudin-1* protein expression in bovine rumen epithelial cells [23]. Additionally, causing impairment of the rumen of the epithelial barrier, long-term high-concentrate diet feeding is also known to lead to excessive apoptosis of rumen epithelium [24]. Apoptosis can be divided into mitochondrial pathways, death receptor pathways, and endoplasmic reticulum pathways [25]. *Apaf-1* is a key molecule in the mitochondrial pathway, which forms an apoptosome with *Cyt-c* [26]. This activates downstream apoptotic proteins, *Caspase-9*, and then further activates other caspases, e.g., *Caspase-3* and *Caspase-7* [26]. *Fas* is the most representative cell death receptor, which can activate downstream apoptotic proteins *Caspase-8* and *Caspase-3/7*. Zhang et al. reported that wine grape pomace contains polyphenols that relieve jejunum epithelial apoptosis by reducing Caspase-8, Caspase-9, and Caspase-3 protein levels in lamb [27]. Supplementation with CEC inhibited rumen epithelial cell apoptosis by decreasing the mRNA expression of the apoptotic mitochondrial pathway (*Apaf-1*, *Cyt-C*, *Caspase-9*, *Caspase-3*, and *Caspase-7*) and the apoptotic death receptor pathway (*Caspase-8*, *Caspase-3*, and *Caspase-7*). *Bcl-2* and *Bax*, major members of the Bcl-2 family, act as anti-apoptotic and pro-apoptotic proteins, respectively [28]. However, our work found that CEC treatment had no significant effect on the mRNA expression of *Bax* and *Bcl-2*. The CEC treatment could alleviate high-concentrate diet-induced apoptosis and improve rumen barrier function.

As reported, low ruminal pH can cause excess deaths of gram-negative bacteria, produce a number of lipopolysaccharides, and then induce rumen inflammation [29]. The molecular mechanisms of inflammation are closely linked with activation of the *TLR4* pathway. Once recognized by *TLR4*, which will promote the production of pro-inflammatory cytokines TNF-α, IL-1β, and IL-6 [30]. Our results revealed that the mRNA expression of *TLR4* and the concentrations of IL-1β, IL-12, and TNF-α were decreased by CEC supplementation. These results agree with Wang et al., who stated that phloretin pretreatment decreased the mRNA expression of *IL-1β*, *IL-6*, *TNF-α*, and *TLR4* in bovine rumen epithelial cells [23]. Moreover, Naringin (bioflavonoid) supplementation also reduced IL-6 and TNF-a levels in the plasma of goats fed a high-concentrate diet [31]. Of particular note, a mixture of trans-cinnamaldehyde and eugenol has been reported to have strong antimicrobial and anti-inflammatory properties [32]. On the basis of the obtained results, we speculated that the anti-inflammation effects of CEC may be responsible for modulating rumen bacterial communities. This finding was also confirmed by the results of high-throughput sequencing, which showed that CEC decreased the population of inflammation-related bacteria (*Escherichia_Shigella* and *Escherichia_coli*).

The rumen microbiota is of crucial importance to rumen health. To further explore the protective effect of CEC on rumen, we therefore examined the effect of CEC on the structure and composition of rumen bacteria by high-throughput sequencing of the 16S rRNA gene V4 region and the relationship between altered rumen bacteria (phylum and genus) and rumen health-related indices by Spearman’s correlation analysis. The PCoA analysis showed a significant difference in bacterial composition between the two groups. Interestingly, supplementation with CEC reduced bacterial richness, which is consistent with the results reported by Díaz Carrasco et al. [33]. It is possible that the relative abundances of Proteobacteria, Enterobacteriaceae, *Escherichia_shigella*, and *Escherichia_coli* were deceased by CEC; thus, the reduced relative abundances of these pathogenic bacteria species may have resulted in a lack of effects on rumen bacterial richness. At the phylum level, lambs given CEC had a greater relative abundance of Firmicutes and Synergistota and a lower abundance of Acidobacteriota, Chloroflexi, and Gemmatimonadota in the present study. De Nardi et al. (2016) found that polyphenols enhanced the abundance of Bacteroidetes, Firmicutes, and Tenericutes in the rumen of heifers fed a high-grain diet [34]. We attribute the difference between our findings and previous reports to differences in phytochemicals as well as animal species. The enrichment in Synergistota could be associated with phenol-degrading [35]. The function of Acidobacteriota, Chloroflexi, and Gemmatimonadota in the rumen is rarely reported. At the genus level, the relative abundance of *Rikenellaceae_RC9_gut_group*, *Olsenella*, *Schwartzia*, *Erysipelotrichaceae_UCG-002*, *Lachnospiraceae_NK3A20_group*, *Acetitomaculum*, *[Eubacterium]_ruminantium_group*, *Prevotellaceae_UCG-004*, *Christensenellaceae_R-7_group*, *Sphaerochaeta*, *Pyramidobacter*, and *[Eubacterium]_eligens_group* were enhanced by CEC treatment. Similarly, Naringin (bioflavonoid) addition also increased the relative abundance of *Rikenellaceae_RC9_gut_group* in the rumen of goats fed a high-concentrate diet [31]. *Rikenellaceae_RC9_gut_group* is a member of the family Rikenellaceae, which is involved in hydrogen production [36]. Meanwhile, endogenous hydrogen can suppress the production of pro-inflammatory cytokines TNF-α, IL-1β, and IL-6 [37]. Perilla frutescens leaf (mainly contains phenolic compounds and flavonoids) increased the relative abundance of *Acetitomaculum* in the rumen of cows, which was consistent with our results [38]. The enrichment of *Olsenella* in the feces of *Artemisia argyi* leaves extract (rich in organic acids and flavonoids)—supplemented mice [39]. Moreover, Olsenella was positively correlated with the anti-inflammatory cytokine IL-10 levels [40]. Our study found that the relative abundances of *Acetitomaculum* and *Olsenella* were negatively correlated with IL-1β concentration, suggesting that the anti-inflammation effects of CEC could be related to modulation of *Rikenellaceae_RC9_gut_group*, *Acetitomaculum,* and Olsenella relative abundance. Additionally, cows fed with dandelion (contains phenolic compounds and flavonoids) were reported to exhibit higher ruminal *Christensenellaceae_R-7_group*, *Prevotellaceae_UCG_003*, and *Lachnospiraceae* spp. abundance, and *Christensenellaceae_R-7_group* was positively correlated with butyrate [41]. A previous study reported that the supply of sodium butyrate reversed the damage to the rumen epithelium tight-junction during SARA [42]. The observed increase in *Christensenellaceae_R-7_group* and positive correlation between *Christensenellaceae_R-7_group* and butyrate concentrations and claudin-4 mRNA expression indicated the impact of CEC intervention on enhancing rumen epithelial barrier function. *Schwartzia* is a succinate-fermenting bacteria [43]. *Eubacterium* spp. were shown to degrade flavonoids in human and rat feces [44]. Furthermore, the LEfSe analysis revealed that the lambs in the CON group enriched differential OTUs most of these OTUs belong to the family Enterobacteriaceae (*Escherichia_Shigella* and *Escherichia_coli*). The best predictor of severe grain-induced SARA was *Escherichia_coli* [45]. However, the lambs in the CEC group enriched *Megasphaera* and *Megasphaera_elsdenii*. *Megasphaera elsdenii* is a lactic acid-consuming bacteria that can convert the lactic acid to propionic acid [46]. The increased *Megasphaera elsdenii* could utilize lactic acid, thereby reducing the risk of lactic acidosis.

## 5. Conclusions

In summary, supplementation with CEC improved rumen health by reducing inflammation and apoptosis, protecting barrier function, and modulating the bacterial community. Thus, CEC could be a promising dietary rumen enhancer to alleviate the negative effects of high-concentrate feeding in intensive ruminant production.

## Figures and Tables

**Table 1:** Ingredient and nutritional composition of the basal diet (air-dry basis).

Items	Content
Ingredient, %	
Maize grain	35.10
Soybean meal (43% crude protein)	5.00
Cottonseed meal	5.00
Corn germ meal	23.00
Sunflower seed shells	13.00
Rice bran meal	12.00
Limestone	1.50
Salt	0.70
Vitamin-mineral premix ^1^	2.00
Dicalcium phosphate	0.70
Bentonite	2.00
Total	100.00
Chemical composition, %	
Metabolizable energy, MJ/kg ^2^	9.63
Crude protein	16.63
Neutral detergent fiber	42.16
Acid detergent fiber	15.06
Ash	10.30
Calcium	1.05
Phosphorous	0.49

^1^ The premix provided the following per kg of diets: VA, 350,000 IU; VD3, 93,750 IU; VE, 938 mg; VK3, 63 mg; VB1, 62 mg; VB2, 188 mg; niacin, 750 mg; pantothenic acid, 500 mg; VB6, 62 mg; biotin, 3.7 mg; folic acid, 38 mg; VB12, 0.7 mg; Se, 18 mg; Zn, 3000 mg; I, 23 mg; Co, 30 mg; Mn, 2500 mg; Fe, 3240 mg; Cu 500 mg. ^2^ Energy was calculated, and others were measured.

**Table 2:** Information on primer sequences.

Gene ^1^	Nucleotide Sequences 5′-3′	Size/bp	GenBank No.
*ZO-1*	F:TTGTAGAATCCGATGTGGG R:CCTGCTGTCTTAGGAAGTGTAT	251	XM_042235170
*Occludin*	F:GGTAACTTGGAGACGCTT	232	XM_015101255
	R:CTGCTTGTAGGCTCTTGTAT		
*Claudin-1*	F:GCTTCATCCTGGCGTTTC	126	NM_001185016
	R:TCCACAGCCCCTCGTAGA		
*Claudin-4*	F:CTTCATCGGCAGCAACAT R:ACAACAGCACGCCAAACA	191	NM_001185017
*Apaf-1*	F:TGGCAGTGGTGGCTTTGT R:ATCACACAATGGACCCAACTTA	106	XM_042247106
*Caspase-3*	F:TGAGATGCTGAAAAAGTACGCT R:CAGAATCGGTGGAAAAGGAC	103	XM_015104559
*Caspase-7*	F:GACAGAAGAACAGGAATGGGTG R:TGGCACAAGAGCAGTCGTTA	118	XM_012102956
*Caspase-8*	F:CTCGGGGATACTGTTTGA R:GCAGTCTTTGGTTTTGTGG	233	XM_012142477
*Caspase-9*	F:AGTTGGACTCGGGTTTTC R:GTCTGTCTGTTGGCATTTCT	179	XM_012187488
*Cyt-C*	F:CTACCTCCGACTCACCGACA R:AGGGGAATCTGCTGACCATC	183	XM_042240814
*Fas*	F:CCAGAGGCATACAGCATCATC R:CATAGGTGTCTTCCCATTCCA	143	NM_001123003
*TLR4*	F:CCGTAAGGTGATTGTCGTGG R:TCCTGTTCAGAAGGCGATAGA	185	NM_001135930
*Bax*	F:CTCACTGCCTCACTCACC R:AGACCACTCCTCCCTACC	173	XM_027978592
*Bcl-2*	F:TTTGATTTCTCCTGGCTGTC R:CTGCTTTCACGAACCTTTTG	142	XM_027960877
*β-actin*	F:CAGCAGATGTGGATCAGCAAGCAG R:TTGTCAAGAAAAAGGGTGTAACGCA	110	XM_042250650

^1^ *ZO-1* = zonula occludens-1; *Apaf-1* = Apoptotic protease activating factor-1; *Cyt-C* = cytochrome c; *TLR4* = toll-like receptor 4; *Bax* = B-cell lymphoma-2 associated X protein; *Bcl-2* = B-cell lymphoma-2.

**Table 3:** Effects of CEC supplementation on the growth performance of lambs.

Items	CON ^1^	CEC ^2^	SEM ^3^	*p*-Value
Initial body weight, kg	30.05	30.16	0.73	0.95
Final body weight, kg	42.04	44.52	0.97	0.24
Average daily weight gain(ADG), g d^−1^	199.86	239.42	8.95	0.02
Dry matter intake (DMI), g d^−1^	1339.60	1339.98	52.41	0.99
DMI/ADG	6.67	5.65	0.29	0.10

^1^ CON = control diet; ^2^ CEC = supplemented with 80 mg/kg blend of cinnamaldehyde, eugenol, and capsicum oleoresin; ^3^ SEM = standard error of the mean.

**Table 4:** Effects of CEC supplementation on the ruminal morphology of lambs.

Items	CON ^1^	CEC ^2^	SEM ^3^	*p*-Value
papillae length (μm)	2368.45	2283.73	66.80	0.56
papillae width (μm)	512.78	469.25	24.84	0.41
muscle layer thickness (μm)	2067.03	1933.33	72.97	0.38
epithelial cell thickness (μm)	133.77	152.98	4.25	0.01

^1^ CON = control diet; ^2^ CEC = supplemented with 80 mg/kg blend of cinnamaldehyde, eugenol, and capsicum oleoresin; ^3^ SEM = standard error of the mean.

**Table 5:** Effects of CEC supplementation on the ruminal fermentation parameters of lambs.

Items	CON ^1^	CEC ^2^	SEM ^3^	*p*-Value
pH	5.22	5.34	0.04	0.21
Ammonia nitrogen (mg/dL)	18.07	10.65	2.00	0.05
Total VFA (mmol/L)	13.53	12.72	0.48	0.42
Individual VFA (% total VFA)				
Acetate	44.31	43.36	0.73	0.54
Propionate	38.62	36.88	0.52	0.10
Butyrate	10.90	13.79	0.73	0.04
Isobutyrate	0.24	0.31	0.04	0.41
Valerate	3.76	3.66	0.30	0.89
Isovalerate	0.27	0.32	0.04	0.60
Acetate: Propionate	1.15	1.18	0.03	0.60

^1^ CON = control diet; ^2^ CEC = supplemented with 80 mg/kg blend of cinnamaldehyde, eugenol, and capsicum oleoresin; ^3^ SEM = standard error of the mean.

**Table 6:** Effects of CEC supplementation on the concentrations of cytokines in rumen epithelial of lambs.

Items	CON ^1^	CEC ^2^	SEM ^3^	*p*-Value
IL-1β (pg/mL)	151.03	100.84	10.62	<0.01
IL-6 (pg/mL)	54.11	51.15	4.24	0.75
IL-12 (pg/mL)	193.93	134.60	16.42	0.05
IL-10 (pg/mL)	140.09	138.85	5.78	0.93
TNF-α (pg/mL)	187.88	146.35	9.66	0.01
INF-γ (pg/mL)	80.14	76.58	2.90	0.59

^1^ CON = control diet; ^2^ CEC = supplemented with 80 mg/kg blend of cinnamaldehyde, eugenol, and capsicum oleoresin; ^3^ SEM = standard error of the mean; IL-1β = interleukin-1β; IL-6 = interleukin-6; IL-12 = interleukin-12; IL-10 = interleukin-10; TNF-α = tumor necrosis factor-α; INF-γ = interferon-γ.

## Data Availability

The original contributions presented in the study are included in the article’s additional files; further inquiries can be directed to the corresponding author. The obtained raw paired-end reads were deposited in the NCBI Sequence Read Archive (Bioproject: PRJNA912559).

## Appendix A

**Table A1:** Effects of CEC supplementation on the ruminal microbiota of lambs (genus level) %.

Items	CON	CEC	*p*-Value
*Prevotella*	24.35 ± 10.37	21.27 ± 5.31	0.54
*Succinivibrio*	10.26 ± 10.29	6.87 ± 3.93	0.48
*Succinivibrionaceae_UCG-001*	11.26 ± 9.64	7.36 ± 4.88	0.41
*Succiniclasticum*	3.41 ± 2.53	5.55 ± 4.30	0.32
*Rikenellaceae_RC9_gut_group*	4.53 ± 2.86	8.38 ± 2.72	0.04
*Treponema*	2.94 ± 4.49	3.11 ± 2.91	0.94
*Prevotellaceae_UCG-001*	3.81 ± 3.22	2.51 ± 1.83	0.42
*Lactobacillus*	1.16 ± 2.38	0.46 ± 0.31	0.50
*Dialister*	1.66 ± 0.48	2.26 ± 1.63	0.42
*Fibrobacter*	1.04 ± 1.73	1.62 ± 0.81	0.48
*Sharpea*	1.80 ± 1.52	0.58 ± 0.41	0.11
*unidentified_Chloroplast*	0.07 ± 0.06	0.70 ± 1.48	0.35
*Erysipelotrichaceae_UCG-002*	0.24 ± 0.36	1.52 ± 0.94	0.02
*Megasphaera*	0.31 ± 0.38	0.91 ± 0.88	0.17
*Lachnospiraceae_NK3A20_group*	0.69 ± 0.30	1.52 ± 0.59	0.02
*Escherichia-Shigella*	0.54 ± 0.84	0.06 ± 0.07	0.23
*Shuttleworthia*	0.54 ± 0.50	0.69 ± 0.10	0.52
*Ruminococcus*	0.71 ± 0.41	1.07 ± 0.26	0.11
*Olsenella*	0.58 ± 0.30	1.10 ± 0.26	0.01
*Syntrophococcus*	0.57 ± 0.37	0.82 ± 0.36	0.27
*[Ruminococcus]_gauvreauii_group*	0.34 ± 0.39	0.70 ± 0.36	0.13
*NK4A214_group*	0.33 ± 0.37	0.21 ± 0.08	0.48
*Catonella*	0.06 ± 0.04	0.36 ± 0.33	0.08
*[Eubacterium]_ruminantium_group*	0.40 ± 0.10	0.63 ± 0.17	0.02
*Saccharofermentans*	0.25 ± 0.34	0.24 ± 0.12	0.92
*Acetitomaculum*	0.22 ± 0.07	0.49 ± 0.24	0.04
*Acidaminococcus*	0.40 ± 0.22	0.61 ± 0.14	0.07
*Prevotellaceae_NK3B31_group*	0.13 ± 0.29	0.00 ± 0.00	0.32
*Prevotellaceae_YAB2003_group*	0.02 ± 0.03	0.18 ± 0.25	0.17
*Bacteroides*	0.16 ± 0.22	0.02 ± 0.01	0.18
*UCG-002*	0.22 ± 0.17	0.23 ± 0.06	0.91
*[Eubacterium]_nodatum_group*	0.19 ± 0.20	0.26 ± 0.05	0.41
*Selenomonas*	0.00 ± 0.00	0.10 ± 0.20	0.29
*Alloprevotella*	0.14 ± 0.18	0.26 ± 0.15	0.26
*Prevotellaceae_UCG-004*	0.13 ± 0.08	0.29 ± 0.09	0.01
*Desulfovibrio*	0.15 ± 0.16	0.25 ± 0.06	0.23
*Lachnospiraceae_NK4A136_group*	0.08 ± 0.16	0.00 ± 0.00	0.27
*Bifidobacterium*	0.15 ± 0.14	0.05 ± 0.04	0.16
*Allisonella*	0.12 ± 0.02	0.19 ± 0.11	0.19
*Erysipelotrichaceae_UCG-006*	0.10 ± 0.11	0.17 ± 0.08	0.24
*U29-B03*	0.06 ± 0.12	0.03 ± 0.02	0.56
*Christensenellaceae_R-7_group*	0.04 ± 0.03	0.16 ± 0.06	<0.01
*Mitsuokella*	0.00 ± 0.00	0.06 ± 0.10	0.26
*unidentified_Mitochondria*	0.18 ± 0.06	0.18 ± 0.07	0.95
*Schwartzia*	0.01 ± 0.01	0.10 ± 0.09	0.05
*Sphaerochaeta*	0.02 ± 0.01	0.15 ± 0.06	<0.01
*Veillonellaceae_UCG-001*	0.05 ± 0.09	0.04 ± 0.02	0.80
*Solobacterium*	0.08 ± 0.07	0.15 ± 0.07	0.09
*Agathobacter*	0.02 ± 0.01	0.08 ± 0.07	0.11
*Erysipelotrichaceae_UCG-007*	0.03 ± 0.06	0.08 ± 0.07	0.23
*Parasutterella*	0.05 ± 0.07	0.00 ± 0.00	0.18
*Pyramidobacter*	0.04 ± 0.02	0.13 ± 0.04	<0.01
*Faecalibaculum*	0.03 ± 0.07	0.00 ± 0.00	0.28
*Kandleria*	0.00 ± 0.00	0.03 ± 0.07	0.26
*Erysipelotrichaceae_UCG-009*	0.05 ± 0.03	0.08 ± 0.05	0.21
*SP3-e08*	0.04 ± 0.03	0.06 ± 0.05	0.35
*UCG-005*	0.04 ± 0.05	0.01 ± 0.01	0.29
*[Eubacterium]_eligens_group*	0.04 ± 0.03	0.10 ± 0.02	<0.01
*MND1*	0.10 ± 0.02	0.00 ± 0.00	<0.01

CON = control diet; CEC = supplemented with 80 mg/kg blend of cinnamaldehyde, eugenol, and capsicum oleoresin.
